# BMI, irAE, and gene expression signatures associate with resistance to immune-checkpoint inhibition and outcomes in renal cell carcinoma

**DOI:** 10.1186/s12967-019-02144-7

**Published:** 2019-11-25

**Authors:** Brian W. Labadie, Ping Liu, Riyue Bao, Michael Crist, Ricardo Fernandes, Laura Ferreira, Scott Graupner, Andrew S. Poklepovic, Ignacio Duran, Saman Maleki Vareki, Arjun V. Balar, Jason J. Luke

**Affiliations:** 1 Department of Internal Medicine, University of Chicago, Chicago, IL USA; 2 Department of Public Health Sciences, University of Chicago, Chicago, IL USA; 3 Hillman Cancer Center, University of Pittsburgh Medical Center, Pittsburgh, PA USA; 4 Division of Hematology/Oncology, University of Pittsburgh, Pittsburgh, PA USA; 5 Division of Hematology/Oncology, New York University School of Medicine and Langone Medical Center, New York, NY USA; 6 Division of Medical Oncology, Schulich School of Medicine & Dentistry, Western University and London Health Sciences Centre, London, ON Canada; 7 Division of Medical Oncology, University Hospital Marques de Valdecilla, Cantabria, Spain; 8 Division of Hematology/Oncology, Virginia Commonwealth University, Richmond, VA USA

**Keywords:** Renal cell carcinoma, Immune-checkpoint inhibition, Immunotherapy, Biomarkers, BMI

## Abstract

**Background:**

Clinical variables may correlate with lack of response to treatment (primary resistance) or clinical benefit in patients with clear cell renal cell carcinoma (ccRCC) treated with anti-programmed death 1/ligand one antibodies.

**Methods:**

In this multi-institutional collaboration, clinical characteristics of patients with primary resistance (defined as progression on initial computed tomography scan) were compared to patients with clinical benefit using Two sample t-test and Chi-square test (or Fisher’s Exact test). The Kaplan–Meier method was used to estimate the distribution of progression-free survival (PFS) and overall survival (OS) in all patients and the subsets of patients with clinical benefit or primary resistance. Cox’s regression model was used to evaluate the correlation between survival endpoints and variables of interest. To explore clinical factors in a larger, independent patient sample, The Cancer Genome Atlas (TCGA) was analyzed. RNAseq gene expression data as well as demographic and clinical information were downloaded for primary tumors of 517 patients included within TCGA-ccRCC.

**Results:**

Of 90 patients, 38 (42.2%) had primary resistance and 52 (57.8%) had clinical benefit. Compared with the cohort of patients with initial benefit, primary resistance was more likely to occur in patients with worse ECOG performance status (p = 0.03), earlier stage at diagnosis (p = 0.04), had no prior nephrectomy (p = 0.04) and no immune-related adverse events (irAE) (p = 0.02). In patients with primary resistance, improved OS was significantly correlated with lower International Metastatic RCC Database Consortium risk score (p = 0.02) and lower neutrophil:lymphocyte ratio (p = 0.04). In patients with clinical benefit, improved PFS was significantly associated with increased BMI (p = 0.007) and irAE occurrence (p = 0.02) while improved OS was significantly correlated with overweight BMI (BMI 25–30; p = 0.03) and no brain metastasis (p = 0.005). The cohort TCGA-ccRCC was examined for the correlations between gene expression patterns, clinical factors, and survival outcomes observing associations of T-cell inflammation and angiogenesis signatures with histologic grade, pathologic stage and OS.

**Conclusions:**

Clinical characteristics including performance status, BMI and occurrence of an irAE associate with outcomes in patients with ccRCC treated with immunotherapy. The inverse association of angiogenesis gene signature with ccRCC histologic grade highlight opportunities for adjuvant combination VEGFR2 tyrosine kinase inhibitor and immune-checkpoint inhibition.

## Background

Renal cell carcinoma is eighth in incidence and mortality among all cancers as measured in the Surveillance, Epidemiology, and End Results database [1]. Clear cell renal cell carcinoma (ccRCC) is the most common and well-studied histologic subtype of RCC and carries the highest risk of metastatic spread [2]. Eighty percent of ccRCC have inactivating mutations in the *Von Hippel Lindau* gene which stabilizes hypoxia inducible factors and leads to overexpression of vascular endothelial growth factor receptor and platelet-derived growth factor receptor which promote angiogenesis, tumor growth and metastasis [3].

Treatment of ccRCC has changed dramatically over a short period of time. Historically ccRCC has been one of a select number of tumors where cytokine therapies such as interleukin-2 or interferon-α have been used to treat metastatic disease [4, 5]. mTOR inhibitors and tyrosine kinase inhibitors (TKI) against VEGF receptor 2 (VEGFR2) developed as disease specific targeted therapies and remain therapeutic standards [6]. More recently immune-checkpoint inhibition (ICI), centered on programmed death-1 (PD-1) or programmed death ligand-1 (PD-L1) as well as cytotoxic T lymphocyte antigen 4 (CTLA4), has become a backbone of therapy. In previously untreated metastatic ccRCC, anti-PD1/L1 antibodies combined with VEGFR2 TKI is emerging [7, 8].

Despite promising activity, many patients have tumors that are refractory to ICI or suffer early progression on treatment. Identifying predictive molecular and clinical markers of resistance is a priority to guide optimal treatment selection. Established biomarkers, such as tumor PD-L1 expression and tumor mutational burden (TMB), have not been demonstrated to have a highly predictive utility in ccRCC [9, 10]. Expression of PD-L1 in ccRCC is somewhat correlated with improved outcomes to ICI however patients with tumors without PD-L1 expression also achieve responses [11–13]. Composite gene expression profiling (GEP) across tumor types has identified gene signatures that associate with treatment response. The T-cell inflamed GEP comprised of IFNγ signaling and T-cell related genes has correlated with treatment response to immunotherapy in multiple tumor types [14–16]. A gene signature of six VEGF-dependent genes validated as a predictive biomarker for anti-VEGF therapy has been used to assess angiogenic activity in ccRCC [17, 18].

Clinical variables may more easily be identified in association with treatment resistance and more favorable outcomes to immunotherapy. ECOG and Karnofsky performance status define functional status of cancer patients and are predictive of outcomes to systemic chemotherapy [19, 20]. The RCC International Metastatic Database Consortium (IDMC) Risk Score defines adverse clinical prognostic risk factors in patients with ccRCC treated with VEGF-targeted therapy [21, 22]. Smoking status and serum albumin correlate with immunotherapy outcomes in some tumors [23]. Increased body-mass index (BMI) was found to correlate with improved outcomes in colorectal and lung cancers as well as immunotherapy response in melanoma and other cancers [24–26]. A prognostic signature incorporating both clinical and genomic variables has been developed in breast cancer [27]. In ccRCC, duration of prior anti-VEGFR2 TKI therapy and neutrophil:lymphocyte ratio (NLR) have been found to be independent predictors of survival [28].

As immunotherapy has become a backbone therapy in the treatment of ccRCC, predictors of lack of response, or primary resistance, are needed. Here we assessed for clinical characteristics that correlate with outcomes in a cohort of patients with ccRCC treated with anti-PD1/PD-L1 and The Cancer Genome Atlas Kidney Renal Clear Cell Carcinoma cohort (TCGA-ccRCC). Recent biomarker analysis found correlation between angiogenesis, T-effector and myeloid inflammatory gene expression and treatment response in ccRCC [18, 29]. VEGF signaling has known immunosuppressive activity and preclinical work has suggested anti-VEGF treatment might enhance efficacy of ICI [30–34]. As such, we interrogated the TCGA cohort with angiogenesis and T-cell inflammation gene signatures and identified clinical, neoplasm histologic grade and stage specific associations that may inform treatment selection and support adjuvant or neo-adjuvant use of ICI and/or VEGFR2-TKI.

## Methods

### Data collection

We performed an international multi-center data collection from patients with stage IV ccRCC who received at least one dose of anti-PD-1/PD-L1 (pembrolizumab, nivolumab, atezolizumab) between 01/01/2011 and 06/01/2018. The participating centers included the University of Chicago Comprehensive Cancer Center (n = 22), Laura and Isaac Perlmutter Comprehensive Cancer Center at NYU Langone (n = 21), Massey Cancer Center at Virginia Commonwealth University (n = 14), London Health Sciences Centre at Western University, Ontario, Canada (n = 17) and Marqués de Valdecilla University Hospital in Santander, Spain (n = 16). Local institutional review board approval, including waiver of consent where appropriate, was obtained at all participating sites using a master data collection protocol.

### Study design

De-identified demographic and clinical variables including but not limited to age, gender, BMI, smoking status, performance status as defined by the Eastern Cooperative Oncology Group (ECOG) were collected. Clinical and laboratory data required for calculation of the RCC IMDC risk score were also collected [21, 22]. Information regarding additional treatments including prior radiation, prior therapies, additional therapies after anti-PD1/L1 treatment and the occurrence of an immune-related adverse event (irAE) were recorded. Clinical outcomes from each center were obtained from their respective electronic medical records with identifiers and dates removed prior to data aggregation.

Radiologic tumor assessment for each patient included computed tomography (CT) scans performed every 8–12 weeks unless otherwise clinically indicated. Patients with either clinical progression or disease progression on first CT evaluation by investigator assessment or death due to cancer prior to first CT evaluation were defined as having primary resistance. Characteristics of patients alive with clinical benefit by investigator assessment on first CT, i.e. those found to not have primary resistance, were then evaluated to identify factors associated with subsequent progression or secondary resistance and survival outcomes.

### Statistical analysis

Baseline demographic data was used to generate descriptive statistics. Tabular summaries were presented for overall patient population and those with primary resistance vs. those with clinical benefit. Continuous variables were summarized using descriptive statistics (n, mean, standard deviation, standard error median, minimum, and maximum). Categorical variables were summarized showing the number and percentage (n, %) of patients within each category. Baseline characteristics between patients with primary resistance and those with clinical benefit were compared using Two sample t-test and Chi-square test (or Fisher’s Exact test).

The Kaplan–Meier method was used to estimate the distribution of progression-free survival (PFS) and overall survival (OS) in all patients and the subsets of patients with clinical benefit or primary resistance. PFS was defined as time from start of treatment to progression by investigator assessment or death from any cause. OS was defined as time from the start of treatment until death from any cause. If no event had occurred, patients were censored at their last follow up visit or 6/30/18, whichever came sooner. Cox’s regression model was used to evaluate the correlation between survival endpoints and the variables of interest. Univariable Cox’s model was first implemented to examine the relationship between survival endpoint and each covariate. Covariates with p-value less than 0.10 were then included in the multivariable models, and a backward selection was performed to derive the final multivariable model for PFS and OS. Statistical analyses were done with SAS 9.4. All statistical tests were two-sided and considered significant at p < 0.05.

### Analysis of TCGA-ccRCC data set

RNAseq gene expression data (release date January 28, 2016) were downloaded from Broad Institute’s GDAC Firehose website [35] for primary tumor of 517 patients from TCGA Kidney RCC (TCGA-ccRCC) database. Harmonized survival data were extracted from the previously published TCGA Pan-Cancer study [36] for progression-free interval event (PFI, defined as “for patient having new tumor event whether it was a progression of disease, local recurrence, distant metastasis, new primary tumors all sites, or died with the cancer without new tumor event, including cases with a new tumor event whose type is N/A” [36]) and OS. Additional demographic and clinical information were downloaded from Genomic Data Commons data portal (GDC) [37]. All tumor samples were used for analysis.

#### Bioinformatics analysis

For the TCGA-ccRCC patients, the RSEM [38]—summarized gene level read counts were upper quartile normalized across all tumor samples and log_2_ transformed. For each tumor, the scores for the T cell-inflamed (Tinfl) or Angiogenesis (Angio) gene signatures were calculated by averaging the expression level of all genes from each signature after centering and scaling across samples for each gene. The probability of survival including PFI and OS were compared between designated groups by log-rank test. The Cox proportional hazard (PH) model was used to evaluate significance of factor of interest in multivariate model with p-values computed by Wald test in function *coxph* from R library survival (v2.43-3).

Gene expression comparison between groups were performed using two-sided Student’s t-test. For multiple comparisons, p-value was adjusted using Benjamini–Hochberg false discovery rate (FDR) correction [39]. Spearman’s correlation ρ was used for measuring statistical dependence between normalized and log_2_-transformed expression level of different gene signatures and was applied in 2 biologically relevant sets: one within non-T cell-inflamed plus intermediate and the other within the T cell-inflamed group. *p* < 0.05 was considered statistically significant. Statistical analysis was performed using R (v3.5.2) and Bioconductor.

## Results

### Baseline patient characteristics

A total of 90 patients treated at four sites in three different countries (USA, Canada, Spain) with PD1/L1 agents were included. Patients were followed for a median of 13.5 months. Pertinent baseline demographic information is reported in Table 1. Mean age was 66 (range 17–92), 27.8% of patients were female. 48.9% of patients had a smoking history. ECOG was 0 or 1 in 85% of patients. 42 of 44 of patients with Stage I–III disease at diagnosis underwent partial or total nephrectomy within 6 months of diagnosis. 42 (46.7%) patients presented with metastatic disease at diagnosis and 27 of 42 (64%) of patients with Stage IV disease at diagnosis underwent nephrectomy.

**Table 1:** Baseline clinical and demographic characteristics

Characteristic	
Median age (range)—year	66 (17–92)
Female gender—no. (%)	25 (27.8%)
Ethnicity—no. (%)	
Caucasian	59 (65%)
African American	8 (9%)
Hispanic	5 (6%)
Asian/Pacific Islander	3 (3%)
Other/unknown	15 (17%)
Smoking history—no. (%)	
No	46 (51%)
BMI status—no. (%)	
Underweight	2 (2%)
Normal	29 (32%)
Overweight	38 (42%)
Obese	21 (23%)
IMDC risk score^a^—no. (%)	
Favorable	18 (20%)
Intermediate	59 (65%)
Poor	6 (6%)
No. of organs with metastasis—no. (%)	
1	8 (9%)
> 2	73 (81%)
Most common sites of metastasis—no. (%)	
Lung	66 (73%)
Spine	31 (34%)
Liver	23 (25%)
Brain	7 (8%)
Nephrectomy—no. (%)	74 (82%)
Previous radiotherapy—no. (%)	52 (58%)
ICI line of treatment—no. (%)	
First line	8 (9%)
Second line	43 (48%)
Third line	28 (31%)
Fourth or more	11 (12%)

^a^Favorable risk responds to an International Renal Cell Carcinoma Database Consortium (IMDC) score of 0, intermediate risk to a score of 1 or 2, poor risk to a score of 3 to 6

The majority of patients were treated off of any investigative protocol except for ten, including three who were treated with pembrolizumab monotherapy, five with pembrolizumab plus indolamine-dioxygenase inhibitor, one with nivolumab plus ipilimumab and one with nivolumab plus an anti-colony stimulating factor receptor agent. ICI as line of therapy included 8.9%, 47.8% or 43% for first, second or third line and beyond, respectively. Prior treatment for metastatic disease for more than 6 months was present in 60% of patients.

### Characteristics of response and post-progression course

Characteristics of response and post-progression course are reported in Table 2. At progression, 31% of patients had new lesions, 29% had growth of existing, 2% had growth of new and existing lesions, and 31% were not recorded. 53 (55%) had additional treatment recorded. Of those patients, 3 pursued hospice. Thirty-one patients (58%) received VEGFR2 TKI monotherapy of which cabozantinib and axitinib were the most commonly used therapies. Eight patients (15%) were placed on an investigative agent in a clinical trial.

**Table 2:** Characteristics of response and progression in all patients

	N (%)
Primary resistance	
Yes	38 (42%)
No	52 (58%)
New or existing lesion at progression	
New	31 (34%)
Existing	29 (32%)
Additional treatment post ICI (n = 53, 58%)	53 (58%)
VEGFR2 TKI containing therapy (n = 39, 74%)	39 (74%)
Cabozantinib	17 (32%)
Axitinib	9 (17%)
VEGFR2 TKI + ICI	6 (11%)
Lenvatinib + everolimus	2 (5%)
Sunitinib	2 (4%)
Lenvatinib	1 (2%)
Pazopanib	1 (2%)
Sorafenib	1 (2%)
Clinical trial, other	8 (15%)
Other (radiation only, hospice)	3 (6%)
Everolimus	2 (4%)
Nivolumab + ipilimumab	1 (2%)
Immune-related adverse events (irAE)	
No	66 (73%)
Type of irAEs	
Colitis	8 (33%)
Pneumonitis	5 (21%)
Hepatitis	3 (13%)
Dermatitis	3 (12%)
irAEs causing treatment discontinuation	
Yes	13 (54%)

Immune-related adverse events (irAE) occurred in 24 (27%) patients. Of those patients, the irAE caused treatment discontinuation in 13 (54%). Colitis was the most common irAE occurring in 8 patients (33%). Pneumonitis and hepatitis occurred in 5 (21%) and 3 (13%) of patients respectively. Pyrexia and fatigue were not routinely recorded. irAE grades were not collected. No deaths were reported related to irAEs.

### Characteristics of patients more likely to experience primary resistance

**Fig. 1:**
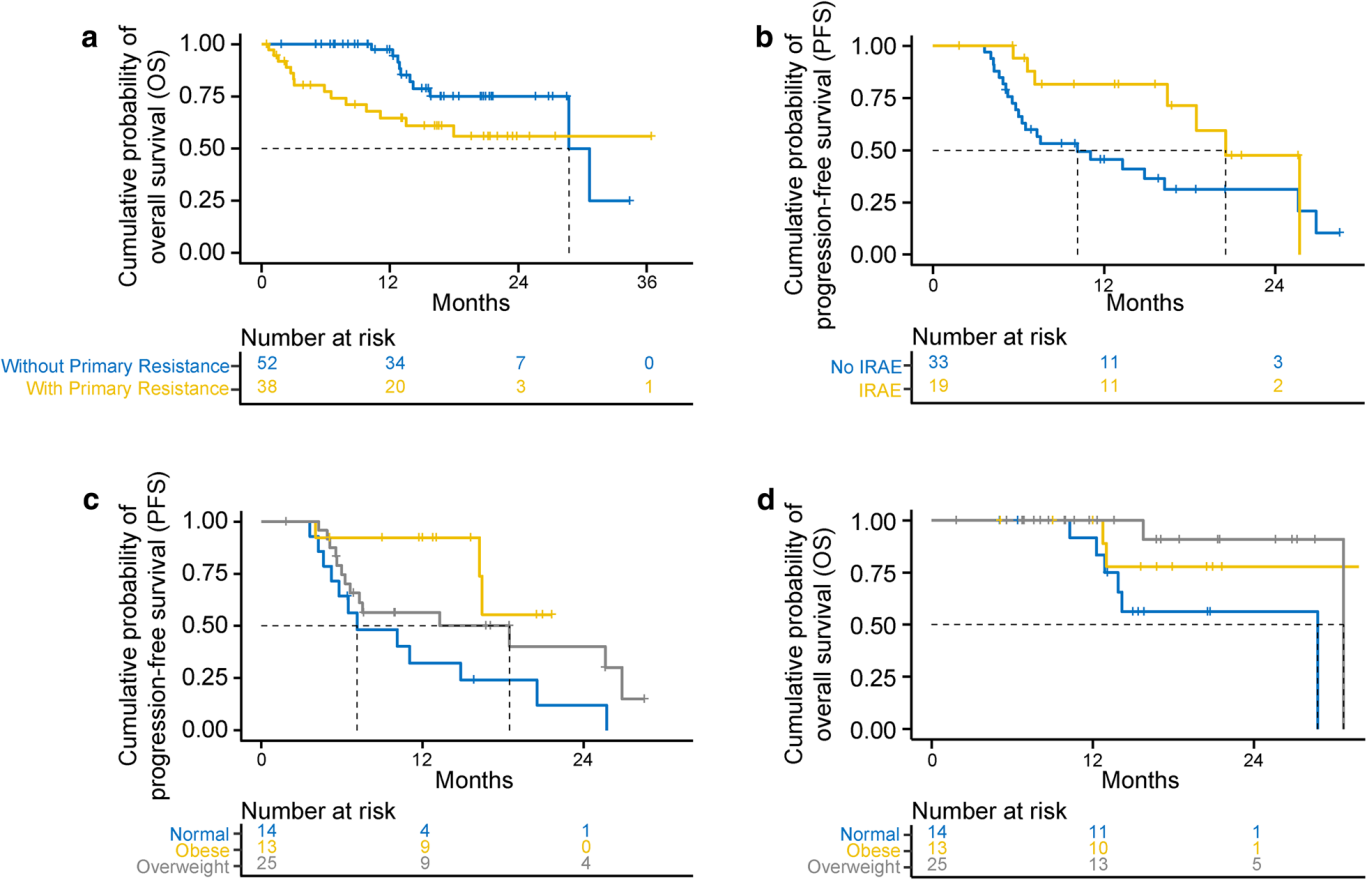
Kaplan-Meier curves depicting survival outcomes. **a** OS in patients by primary resistance status. **b** PFS in patients by irAE occurrence. **c** PFS in patients by BMI status. **d** OS in patients by BMI status

Of 90 patients studied, 38 (42%) patients had primary resistance and 52 (58%) of patients did not. By t-test and Chi squared tests, patients with primary resistance were more likely to have a worse ECOG performance status at the start of immunotherapy (p = 0.03), have an earlier stage at diagnosis (p = 0.04), have not undergone nephrectomy (p = 0.04) and not experience an immune-related adverse event (p = 0.02). We failed to observe any other significant difference between two patient cohorts with respect to other demographic, histologic, prior treatment or biochemical characteristics. Patients who experienced primary resistance had worse OS than those with clinical benefit (Fig. 1a).

### Survival outcomes in patients with clinical benefit

Multivariable analysis of variables associated with improved outcomes in patients who experienced clinical benefit is presented in Table 3. Factors associated with improved PFS were an occurrence of an irAE (p = 0.02) and increased BMI (p = 0.007) (Fig. 1b, c). No association was observed between PFS and the type of irAE experienced. Improved OS was correlated with overweight BMI (p = 0.03), defined as BMI 25–30, and the absence of brain metastasis (p = 0.005) (Table 4). There was a trend towards improved OS in obese patients, but this did not reach statistical significance (p = 0.07), perhaps due to small sample size. In these patients, ECOG PS, IMDC Risk Score, laboratory parameters such as albumin and NLR did not show correlation with PFS or OS. Higher BMI did not show correlation with the likelihood of experiencing an irAE.

**Table 3:** Variables associated with PFS in patients with clinical benefit

	N	Median PFS (in months)	Univariable Analysis		Multivariable Analysis	
			HR (95% CI)	p-value	HR (95% CI)	p-value
Age	52	16.24	0.95 (0.91, 1.00)	0.06		
BMI	52	16.24	0.90 (0.82, 0.98)	0.02	0.87 (0.79, 0.96)	0.007
Smoking history						
No	27	13.28	1.00	Ref		
Yes	25	18.48	0.80 (0.39, 1.67)	0.55		
Nephrectomy						
Total	44	16.24	Ref	Ref		
Partial	3	–	0.31 (0.04, 2.45)	0.27		
None	5	18.48	0.83 (0.25, 2.78)	0.76		
ICI line of treatment						
First line	5	–	1.00	Ref		
Second line	23	18.48	1.73 (0.37, 8.08)	0.49		
Third line	17	13.28	2.65 (0.54, 13.04)	0.23		
Fourth line	5	20.52	2.08 (0.32, 13.56)	0.44		
> 4 prior treatments	2	25.71	0.83 (0.07, 9.45)	0.88		
ECOG PS at start of immunotherapy						
0	19	16.24	1.00	Ref		
1 or 2 or 3	32	18.48	0.83 (0.38, 1.79)	0.63		
Missing	1	–	–	–		
IMDC risk score						
Favorable	13	16.44	1.00	Ref		
Intermediate	32	14.83	1.43 (0.58, 3.53)	0.44		
Poor	3	26.86	0.29 (0.04, 2.38)	0.25		
Occurrence of irAE						
No	33	10.13	1.00	Ref	1.00	Ref
Yes	19	20.52	0.42 (0.17, 0.99)	0.04	0.33 (0.13, 0.82)	0.02

**Table 4:** Variables associated with OS in patients with clinical benefit

IMDC risk score	N	Median OS (in months)	Univariable analysis		Multivariable analysis	
			HR (95% CI)	p-value	HR (95% CI)	p-value
Age	52	28.73	1.00 (0.93, 1.08)	0.94		
BMI group						
Normal	14	28.73	1.00	Ref	1.00	Ref
Overweight	25	30.67	0.19 (0.04, 1.01)	0.05	0.15 (0.03, 0.86)	0.03
Obese	13	–	0.25 (0.04, 1.41)	0.12	0.19 (0.03, 1.11)	0.07
Smoking history						
No	27	30.67	1.00	Ref		
Yes	25	28.73	0.51 (0.14, 1.82)	0.30		
Nephrectomy						
Total	44	28.73	Ref	Ref		
Partial	3	–	–	0.99		
None	5	–	1.25 (0.15, 10.16)	0.84		
ICI line of treatment						
First line	5	–	–	0.99		
Second line	23	30.67	1.00	Ref		
Third line	17	–	0.86 (0.21, 3.64)	0.84		
Fourth line	5	–	0.88 (0.10, 7.67)	0.91		
> 4 prior treatments	2	28.73	1.55 (0.15, 15.97)	0.71		
ECOG PS at start of immunotherapy						
0	19	30.67	1.00	Ref		
1 or 2 or 3	32	28.73	5.96 (0.72, 49.62)	0.10		
Missing	1	–	–	–		
IMDC risk score						
Favorable	13	30.67	1.00	Ref		
Intermediate	32	28.73	1.02 (0.25, 4.13)	0.97		
Poor	3	–	–	0.99		
Occurrence of irAE						
No	33	30.67	1.00	Ref		
Yes	19	28.73	0.80 (0.20, 3.19)	0.75		
Prior brain mets						
No	47	30.67	1.00	Ref	1.00	Ref
Yes	5	15.75	6.49 (1.53, 27.56)	0.01	9.41 (1.94, 45.71)	0.005

### Survival outcomes in patients with primary resistance

Variables associated with overall survival in patients with primary resistance is represented in Table 5. Patients with intermediate IMDC risk score [1, 2] had improved OS compared with those with poor IMDC risk score (> 3) (p = 0.02). Patients with higher pre-treatment NLR had decreased OS (p = 0.04). BMI, smoking history or ECOG performance status did not correlate with survival in these patients.

**Table 5:** Variables associated with OS in patients with primary resistance

Variable	N	Median OS (in months)	Hazard ratio (95% CI)	p-value
IMDC score				
Favorable	5	–	–	–
Intermediate	27	–	0.14 (0.03, 0.73)	0.02
Poor	3	2.99	1.00	Ref
N/A	3	6.51	0.51 (0.06, 4.50)	0.54
Pre-treatment NLR	27	13.51	1.17 (1.00, 1.36)	0.04

### Survival outcomes in all patients

In all 90 patients in the cohort, we observed significant correlation between PFS and experiencing clinical benefit and BMI status (Additional file 1: Table S1). OS was correlated with primary resistance status, BMI (or BMI group), ECOG PS at start of immunotherapy and albumin (Additional file 1: Table S2).

### TCGA ccRCC Dataset Analysis

**Fig. 2:**
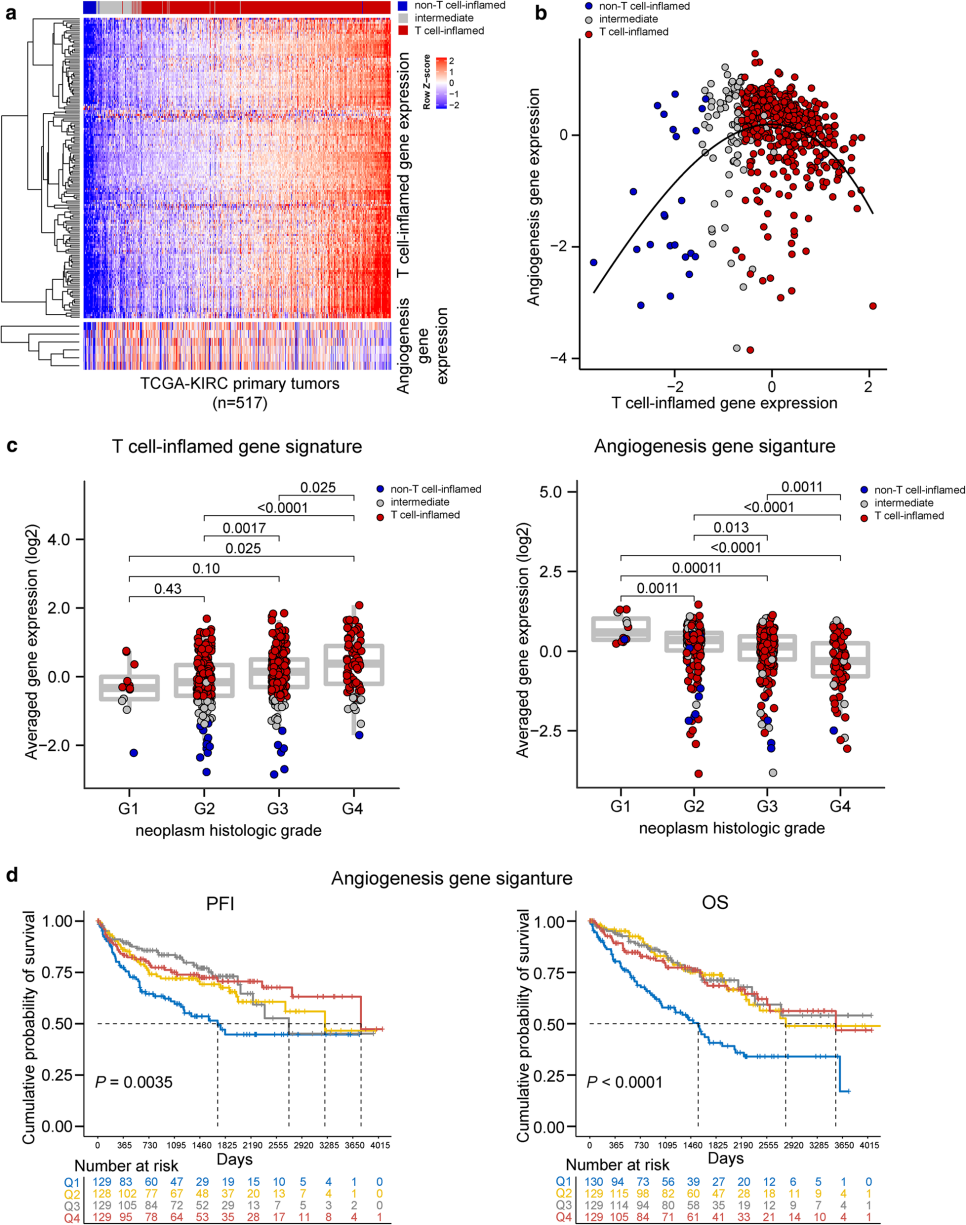
Landscape of T cell-inflamed and angiogenesis gene signature expression and association with survival in TCGA-ccRCC cohort (*n *= 517). **a** Primary tumor samples were categorized into non-T cell-inflamed (blue), T cell-inflamed (red), and intermediate (grey) group based on the expression of T cell-inflamed gene signature. Tumors are shown on the column, and sorted from low to high T cell-inflamed gene expression (left to right). Upper heatmap: T cell-inflamed gene expression; bottom heatmap: angiogenesis gene expression. Gene clusters are shown as dendrogram to the left of each heatmap. **b** Correlation of T cell-inflamed and angiogenesis gene signature expression in tumor groups. **c** Expression of T cell-inflamed and angiogenesis gene signatures in neoplasm histologic grade I to IV. Grade X samples were excluded (*n *= 4). **e** Comparison of survival distributions between patient groups split by quartiles of angiogenesis gene signature expression. PFI = progression-free interval event. OS = overall survival. Q1 to Q4 = expression quartile 1 to 4. Student’s *t*-test was used in **c**, followed by BH-FDR correction for multiple testing. Log-rank test was used in **d**

Using a defined T cell-inflamed gene expression signature (Tinfl [40]), we categorized primary tumors of 517 patients from the TCGA-ccRCC database into non-T cell-inflamed (*n *= 24), T cell-inflamed (*n *= 419), and intermediate (*n *= 74) groups following previously described strategies [40]. For each patient, we calculated expression level of the Tinfl signature and an angiogenesis gene signature (Angio [17]) and correlated the two signatures across all tumor samples (Fig. 2a). Within tumors of lower T cell-inflamed gene expression (Fig. 2b, non-T cell-inflamed and intermediate), Tinfl and Angio signatures are positively correlated (Spearman’s ρ = 0.28, *p *= 0.006) however at higher levels of T cell-inflammation, tumors demonstrate anti-correlation between the two signatures (Spearman’s ρ = − 0.35, *p* = 1.28e−13) (Fig. 2b). Among the demographic and clinical variables investigated in this study, histologic grade of tumors showed an increase of T cell-inflammation and decrease of angiogenesis signature from grade I to IV (FDR-adjusted *p* < 0.05, two-sided Student’s *t*-test) (Fig. 2c). There was a trend to increasing T-cell inflammation signature in later stage disease and more dramatic differences were observed in stage IV relative to stage I (FDR-adjusted *p *< 0.001). Race showed marginally significant differences for Tinfl (*p *= 0.034, two-sided Student’s *t*-test) and Angio (*p *= 0.069) expression with medium effect size [41, 42] (Cohen’s *d* = 0.50 for both signatures). Other variables were not significant for either signature.

We also investigated the association between the gene signatures and patients’ survival by splitting patients into four groups based on quartiles of each gene signature. Angio-Q1 patients showed significantly lower PFI and OS compared to Angio-Q2/3/4 (Fig. 2d), and remained significant after adjusting for age and gender. No significant differences in PFI or OS were observed between Tinfl-Q1/2/3/4 patients. Gender is significantly associated with PFI but not OS, with females showing better PFI compared to males (*p *= 0.012, log-rank test). Age at diagnosis was significantly associated with OS but not PFI, with patients of ≤ 65 years old showing improved OS compared to those of > 65 years old (*p *= 0.0088, log-rank test). Significant prognostic clinical variables included pathologic stage (PFI, OS), neoplasm histologic grade (PFI, OS), white cell count (PFI), platelet quality (PFI, OS), hemoglobin (PFI, OS), consistent with previously known clinical reports. BMI was not investigated for TCGA-ccRCC cohort due to lack of record in height and weight per patient for the calculation of BMI.

## Discussion

Treatment with immune-checkpoint inhibition (ICI) has changed the treatment paradigm in ccRCC however many do not respond to these treatments and no reliable molecular biomarker exists to predict response to ICI in individual patients. PD-L1 immunohistochemistry and TMB have emerged as relevant biomarkers for ICI across many tumors however have not been relevant in ccRCC. Data across tumor types however suggests that some clinical features associate strongly with clinical outcomes to ICI. This study reviewed the course of 90 patients with ccRCC treated with anti-PD1/L1 and identified factors associated with primary resistance including a worse ECOG performance status at the start of ICI, an earlier stage at diagnosis, no prior nephrectomy and no occurrence of an irAE. Overall survival in these patients was correlated with IMDC Risk Score and pre-treatment NLR. In patients with initial benefit, increased BMI and overweight BMI status correlated with improved progression free and overall survival, respectively. Occurrence of an irAE correlated with longer time to progression while brain metastasis correlated decreased OS.

Higher BMI has correlated with a survival advantage to ICI in melanoma and other cancers [24–26, 43]. A recent study demonstrated a survival benefit in patients with higher BMI in ccRCC treated with ICI [44]. The mechanisms by which BMI impact clinical outcomes remain poorly understood. Lalani et al. did not find differences in genomic alteration frequency or tumor mutational burden by BMI status. Hyperadiposity may drive a tumorigenic immune-dysfunction that is more effectively reversed by ICI [45, 46]. However, BMI may not adequately reflect the complexities of body composition. Computerized tomography-based body composition (CTBC) and bioelectrical impedance analysis have defined phenotypes which correlate with outcomes such as high visceral adipose tissue, skeletal muscle density, and sarcopenia [47–49]. Further studies are needed to characterize mechanisms by which these phenotypes overlay with known biomarkers.

Our study demonstrated increased likelihood of response to ICI and improved PFS in patients who experienced and irAE, consistent observations in multiple solid tumors [50, 51]. Studies have demonstrated association between outcomes and incidence of vitiligo and dermatitis in patients with melanoma as well as thyroiditis in NSCLC [52–55]. No specific irAE were associated with improved outcomes in this study. Mechanisms by which irAEs correlate to tumor regression need to be further clarified. One proposed mechanism is cross reactivity of activated T-cells against antigens specific to both tumors and normal tissue, known as antigen sharing [56].

Tissue based biomarkers for ICI across tumor types and especially in ccRCC are evolving. Exploratory biomarkers, such as gene expression profiling suggest that it may be possible to identify sub-populations of patients most likely to benefit to particular treatments. Angiogenesis, T-effector gene (similar to T cell-inflamed) expression signatures associated with outcomes in a recent clinical trial in ccRCC [18, 29]. The Angio^low^ and T-eff^high^ GEP subgroups had improved outcomes to ICI whereas the Angio^high^ subgroup had worse outcomes to ICI but improved outcomes to VEGFR2 TKI. Our analysis of the TCGA revealed an inverse correlation between angiogenesis and T-cell inflammation signatures in tumors of high T cell-inflamed gene expression, a pattern not observed in non-T cell-inflamed tumors. An inverse association between the angiogenesis signature and histologic grade was demonstrated and a positive association between the T-cell inflammation signature and pathologic stage. This data suggests a suppressive role of angiogenesis on T cell-inflammation and may support further development of VEGFR2-TKI in combination or sequential therapy with ICI in earlier stage ccRCC. In clinical trials in non-metastatic ccRCC, perioperative systemic treatment with VEGFR2-TKI was not shown to increase overall survival versus surgery alone [57–59]. Benefit from ICI in the adjuvant and neoadjuvant setting has been observed in multiple cancers including NSCLC, breast cancer and melanoma and multiple phase III clinical trials evaluating ICI in ccRCC in both adjuvant and neoadjuvant settings are ongoing [60–64] (NCT03024996, NCT03142334, NCT03055013).

Our analysis of the TCGA revealed a positive correlation between T-cell inflammation signature and pathologic stage and in our ICI cohort, patients diagnosed at earlier stage were more likely to experience primary resistance to ICI. It should be noted that patients diagnosed at an earlier stage likely received ICI at time of metastatic recurrence in which there was indeed a longer time from initial diagnosis to treatment than those diagnosed at Stage IV disease (60 mo vs. 3 mo, *p* < 0.0001). This latency may account for the increased likelihood of resistance. Perhaps metastatic recurrences progress predominately from tumors diagnosed de-novo and recur in non-inflamed, immunosuppressed or immune-exhausted environments. Investigation of primary ccRCC and ccRCC lung metastases demonstrated differential expressions of immunosuppressive molecules between primary and metastatic tumors [65]. Further work to define the immune microenvironment of metastatic recurrences is warranted.

Patients who did not undergo nephrectomy were also more likely to suffer primary resistance. Pre-clinical work has suggested the primary tumor may produce T-cell inhibitory cytokines that divert antitumor immune response away from metastasis [66]. Correlation between the morphologic immune character of the resected primary tumor, such as Teff/Treg ratio, with outcomes to ICI in ccRCC has previously been demonstrated [67]. A recent study found increased response rate in patients who underwent cytoreductive nephrectomy or metastasectomy while receiving ICI [68]. These conclusions highlight the need to further investigate the correlation between the immune character of the resected tumor and treatment outcomes.

While our study sheds light on factors associated with treatment response to immunotherapy and survival in ccRCC, we acknowledge limitations. This is a retrospective study and while we included all identified patients at each institution, a selection bias cannot be excluded. While we aggregated data across 5 centers internationally, we acknowledge that the sample size could be larger which could expand our potential findings. While our report predominately focused on response, we note that our follow-up time potentially did not fully assess long-term survival outcomes in patients receiving immunotherapy. Finally, the TCGA dataset did not record BMI and these patients were not treated with immunotherapy. We alternatively employed gene expression signatures which have been strongly associated with treatment outcomes however this narrows the conclusions that could be reached.

## Conclusions

This international, multi-institutional effort supports conclusions that clinical factors, notably BMI and occurrence of an irAE, strongly associate with treatment outcomes in patients with ccRCC treated with ICI. We have identified novel predictors as well as variables that support previous studies, all of which may help guide clinical selection criteria for immunotherapy treatment. On the gene expression level we identified biologically relevant gene signatures including the T-cell inflammation and angiogenesis signatures that associate with histologic grade, pathologic stage and survival. Given the suppressive role of angiogenesis on T cell-inflammation, these data may support further development of VEGFR2-TKI in combination or sequential therapy with ICI in earlier stage ccRCC emphasizing the importance of adjuvant and neo-adjuvant strategies.

## Supplementary information

**Additional file 1: Table S1.** Variables associated with PFS in all patients (N = 90). **Table S2.** Variables associated with OS in all patients (N = 90).

## Data Availability

The datasets generated and/or analyzed during the current study are not publicly available due to personal health information protection standards but are available from the corresponding author on reasonable request. TCGA datasets are publicly available on Broad Institute’s GDAC Firehose website, GDC data portal, and relevant publications as described in “Methods”.
