# Supplementary material for: BMI, irAE, and gene expression signatures associate with resistance to immune-checkpoint inhibition and outcomes in renal cell carcinoma
Source: J Transl Med. 2019 Nov 25;17:386. doi: 10.1186/s12967-019-02144-7 (PMC6878694; doi:10.1186/s12967-019-02144-7)
Supplement: Supplementary file 1 — Additional file 1: Table S1. Variables associated with PFS in all patients (N = 90). Table S2. Variables associated with OS in all patients (N = 90). [file 12967_2019_2144_MOESM1_ESM.docx]

**Table S1: Compare PFS in all patients (N=90)**

| Variable |  | N | Median PFS (in months) | Hazard Ratio (95% CI) | p-value |
| --- | --- | --- | --- | --- | --- |
| Primary Resistance | No | 52 | 16.24 | 1.00 | ref |
|  | Yes | 38 | 3.09 | 5.44 (3.18, 9.31) | <0.0001 |
| BMI |  | 90 | 6.61 | 0.93 (0.88, 0.99) | 0.02 |
| BMI group | Underweight | 2 | 2.86 | 3.03 (0.40, 23.25) | 0.29 |
|  | Normal | 29 | 4.24 | 1.00 | ref |
|  | Overweight | 38 | 7.53 | 0.50 (0.28, 0.88) | 0.02 |
|  | Obese | 21 | 16.44 | 0.39 (0.19, 0.82) | 0.01 |
| ECOG PS at start of immunotherapy | 0 | 26 | 7.53 | 1.00 | ref |
|  | 1 | 51 | 5.79 | 1.23 (0.70, 2.17) | 0.48 |
|  | 2 or 3 | 12 | 18.77 | 0.85 (0.33, 2.15) | 0.72 |
|  | NA | 1 | --- | --- | --- |
| Albumin |  | 88 | 7.13 | 0.73 (0.40, 1.33) | 0.30 |
| Hgb |  | 88 | 7.13 | 0.97 (0.85, 1.12) | 0.71 |
| Calcium |  | 88 | 7.13 | 1.17 (0.90, 1.51) | 0.25 |
| Log(ANC) |  | 88 | 7.13 | 0.93 (0.58, 1.48) | 0.74 |
| Lesion at progression | Existing | 32 | 8.28 | 1.00 | ref |
|  | New | 30 | 4.80 | 1.46 (0.85, 2.53) | 0.17 |
|  | NA | 28 | --- | --- | --- |

**Table S2: Compare OS in all patients (N=90)**

| Variable | Level | N | Median OS (in months) | Hazard Ratio (95% CI) | p-value |
| --- | --- | --- | --- | --- | --- |
| Primary Resistance | No | 52 | 28.73 | 1.00 | ref |
|  | Yes | 38 | --- | 2.31 (1.02, 5.21) | 0.04 |
| BMI |  | 90 | 30.67 | 0.91 (0.83, 0.99) | 0.04 |
| BMI group | Underweight | 2 | 13.51 | 2.03 (0.26, 15.81) | 0.50 |
|  | Normal | 29 | 28.73 | 1.00 | ref |
|  | Overweight | 38 | 30.67 | 0.38 (0.15, 0.97) | 0.04 |
|  | Obese | 21 | --- | 0.28 (0.09, 0.91) | 0.03 |
| ECOG PS at start of immunotherapy | 0 | 26 | 30.67 | 1.00 | ref |
|  | 1 | 51 | 28.73 | 4.10 (1.19, 14.13) | 0.03 |
|  | 2 or 3 | 12 | --- | 3.60 (0.71, 18.24) | 0.12 |
|  | NA | 1 | --- | --- | --- |
| Albumin |  | 88 | 30.67 | 0.35 (0.14, 0.84) | 0.02 |
| Hgb |  | 88 | 30.67 | 0.82 (0.67, 1.02) | 0.07 |
| Calcium |  | 88 | 30.67 | 1.42 (0.92, 2.18) | 0.11 |
| Log(ANC) |  | 88 | 30.67 | 1.14 (0.49, 2.62) | 0.77 |
| Lesion at progression | Existing | 32 | 30.67 | 1.00 | ref |
|  | New | 30 | 28.73 | 1.78 (0.70, 4.57) | 0.23 |
|  | NA | 28 | --- | --- | --- |
